# Factors associated with deaths from dengue in a city in a metropolitan region in Southeastern Brazil: a case-control study

**DOI:** 10.1590/0037-8682-0043-2022

**Published:** 2022-09-26

**Authors:** Selma Costa de Sousa, Thais Almeida Marques da Silva, Aleida Nazareth Soares, Mariângela Carneiro, David Soeiro Barbosa, Juliana Maria Trindade Bezerra

**Affiliations:** 1 Universidade Federal de Minas Gerais, Instituto de Ciências Biológicas, Programa de Pós-Graduação em Parasitologia, Belo Horizonte, MG, Brasil.; 2 Faculdade Santa Casa BH, Programa de Pós-Graduação Stricto Sensu em Medicina-Biomedicina, Belo Horizonte, MG, Brasil.; 3 Universidade Federal de Minas Gerais, Faculdade de Medicina, Programa de Pós-Graduação em Infectologia e Medicina Tropical, Belo Horizonte, MG, Brasil.; 4 Universidade Federal de Ouro Preto, Núcleo de Pesquisas em Ciências Biológicas, Programa de Pós-Graduação em Ciências Biológicas, Ouro Preto, MG, Brasil.; 5 Universidade Estadual do Maranhão, Centro de Estudos Superiores de Lago da Pedra, Curso de Licenciatura em Ciências Biológicas, Lago da Pedra, MA, Brasil.; 6 Universidade Estadual do Maranhão, Centro de Ciências Agrárias, Programa de Pós-Graduação em Ciência Animal, São Luís, MA, Brasil.

**Keywords:** Dengue, Severe dengue, Death, Arbovirus infections

## Abstract

**Background:**

Dengue is a public health problem in Brazil. Therefore, this study aimed to analyze factors associated with deaths from dengue in residents of the municipality of Contagem, Metropolitan Region of Belo Horizonte, state of Minas Gerais, Brazil, during the 2016 epidemic.

**Methods:**

To determine the factors associated with deaths due to dengue, we used a logistic regression model (univariate and multivariable) in which the response variable (outcome) was death due to dengue. Independent variables analyzed included demographic variables and those related to symptoms, treatment, hospitalization, testing, comorbidities, and case history.

**Results:**

The factors associated with dengue deaths in the final multivariable model [p < 0.05; 95% confidence interval (CI)] were age (OR = 1.07; 95%CI 1.03-1.11) and presence of bleeding (OR = 8.55; 95%CI 1.21-59.92).

**Conclusions:**

The results showed that age and the presence of bleeding factors increased the risk of dengue death. These findings indicate that warning signs of dengue should be routinely monitored, and patients should be instructed to seek medical attention when they occur. It is also emphasized that the parameters and epidemiological conditions of dengue patients need to be continuously investigated to avoid a fatal outcome.

## INTRODUCTION

Dengue is caused by the Dengue virus (DENV), which belongs to the family *Flaviviridae* and the genus *Flavivirus* ^1^. There are four serotypes (DENV-1, DENV-2, DENV-3, and DENV-4), and in 2013, a fifth serotype was isolated and associated with a severe case of dengue in humans^2^. DENV is also classified as an arbovirus because it is transmitted by arthropod vectors of the genus *Aedes*, such as *Aedes aegypti* (Linnaeus, 1762) (Diptera: Culicidae)^3^, the primary vector. This virus can infect various types of cells, including the liver, spleen, brain, and other vital organs^1^.

The severity of arbovirus depends on several factors, such as extremes in age groups, comorbidities, access to healthcare, and low income. Advanced age is considered a risk factor for developing severe dengue. People over 65 years of age have a higher prevalence of comorbidities than younger age groups, and children under 15 years of age, especially those under one year, are more likely to be affected by severe forms of the disease^4^.

Access to healthcare, mediated by other factors such as income and poverty, as well as limited access to healthcare facilities (which may be related to the education of the individual and the healthcare team), may also contribute to fatal dengue illness. When individuals are well counseled by health professionals, they seek service on time, leading to better diagnosis, appropriate treatment, and consequently low mortality rates. Therefore, adopting disease prevention strategies and identifying the occurrence of deaths are extremely important in preventing new deaths from dengue^5^.

The year 2016 was characterized by a large dengue epidemic in Brazil, with 1,500,535 cases of the disease reported. The southeastern region (85,273 cases; 57.1%) had the highest number of probable cases of the disease^6^. The state of Minas Gerais reported 517,830 cases, of which 10,602 were hospitalized for dengue and severe dengue. Of the hospitalized cases, 10,501 were urgent, and 101 were elective. A total of 520 people died, 283 from dengue and 237 from other causes^7^. In the municipality of Contagem, 44,341 cases of arbovirus (8.5% of them in the state of Minas Gerais) were reported in 2016, with 30 deaths (17 deaths from the disease and 13 from other causes)^8^.

This study aimed to analyze factors associated with dengue deaths among residents of the municipality of Contagem, in the Metropolitan Region of Belo Horizonte, state of Minas Gerais, Brazil, during the 2016 epidemic.

## METHODS

### Study Area

The municipality of Contagem is located in the metropolitan region of Belo Horizonte, Minas Gerais. The city has important industrial centers, such as the Cidade Industrial Center, founded in 1941^9^ ^,^ ^10^. This municipality is part of the polarizing core of urban and economic activity in the Metropolitan Region of Belo Horizonte (MRBH). It has an area of 195,268 km^2^, a density of 3,375 inhabitants/km^2^, an altitude of 858 m, and a tropical climate. The estimated population of the municipality in 2021 was 673,849 inhabitants^9^.

The municipality was divided into eight administrative regions. The municipal health network in Contagem consists of a municipal maternity ward, a municipal hospital, 23 basic health units, 87 family health teams, and 4 immediate care units or emergency care units, as they are now called: Low and Medium Complexity Emergency Department, the *Pronto-Socorro Geraldo Pinto Vieira*; a *Centro de Consultas Especializadas Iria Diniz*; a Reference Service for Women's Health and one for Workers' Health; a Center for Psychosocial Care; a Reference Center for Child and Adolescent Care; a Reference Center for Children and Adolescents; and 10 Oral Health Teams. Of the eight health districts, four had an emergency response unit^10^.

### Study design and sample size

This was a case-control study of patients admitted to *Hospital Municipal de Contagem* (HMC) with suspected dengue infection between January 1 and December 31, 2016. HMC is a tertiary hospital that admits patients from the Unified Health System (*Sistema Único de Saúde* - SUS) in Contagem, as well as patients referred by the SUSFácil MG with a probable dengue diagnosis for hospital treatment. Therefore, the following cases were considered: (a) case definition: dengue deaths that occurred in residents of the municipality of Contagem during the 2016 epidemic year and were investigated by the Municipal Health Department (*Secretaria Municipal de Saúde* - SMS) of this city; and (b) control definition: probable cases of dengue in residents of the municipality of Contagem in 2016 that required hospitalization and progressed to cure. These patients were identified through the SUSFácil MG, and those hospitalized in HMC in the municipality of Contagem were selected. Data were then collected from the selected medical records.

All deaths that occurred during the study period were included in the study. For each case (dengue as the underlying cause of death), three controls were selected (patients diagnosed with dengue and cured), making the sample a total of 75 individuals (19 cases and 56 controls). Considering the number of cases and controls, the significance of the test was calculated using the OpenEpi^11^ software. The frequency of exposure in these cases was based on a study by Campos et al. (2015)^12^, and a 95% confidence interval (95%CI) was used as a parameter; the estimated exposure ranged from 50% to 70% in cases and from 25% to 35% in controls. The estimated power ranged from 64.8% to 77.1%.

Banks were pooled and qualified after analyzing hospital admissions and death certificates. For the controls (admissions for dengue), of the 66 admissions, ten patients were excluded: four because they were not residents, four because they were diagnosed with another disease, and two because they were deceased, for a total of 56 people. Regarding cases (deaths), 19 deaths were identified among Contagem residents.

### Study population

According to the SUSFácil MG report on hospitalizations for dengue, there were 157 probable hospitalizations for dengue among Contagem residents in 2016. Of these, 89 (56.7%) were admitted to HMC. The following filters were used as search criteria for patient selection: (a) report of admissions by hospital facility; (b) patient's city: count; (c) diagnoses: A90 classical dengue fever, A91 dengue hemorrhagic fever; and (d) period from January 1 to December 31, 2016.

**FIGURE 1:**
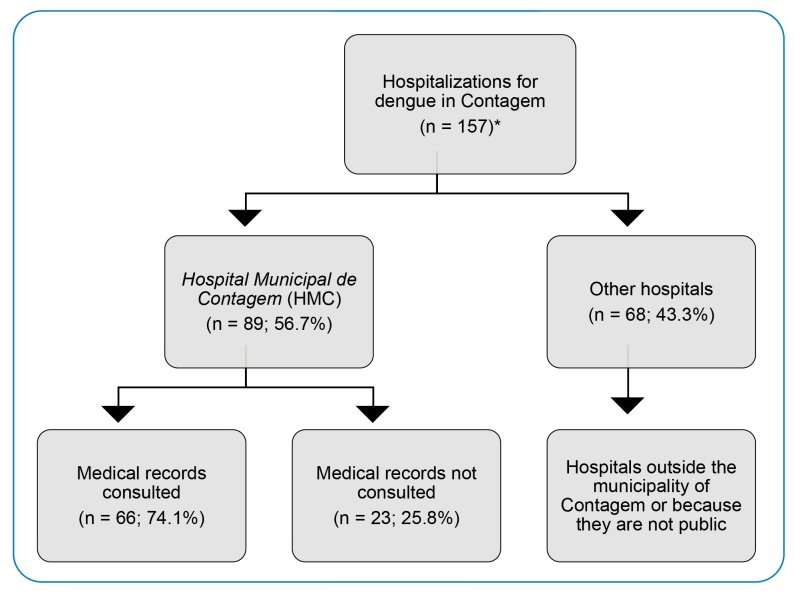
Flowchart of the analyzed medical records of the population included in the study on factors associated with death from dengue in 2016 in residents of the municipality of Contagem, State of Minas Gerais, Brazil. Source: *Data from SUSFácil MG collected on July 17, 2019. **n:** number of individuals.

The report provided by SUSFácil MG informs about all hospitalizations performed in the state of Minas Gerais for suspected dengue; therefore, the request for hospitalization does not require a confirmatory test but only a suspicion with compatible symptoms. Of the 89 patients admitted to HMC, information was obtained from 66 (74.2%) medical records. Twenty-three medical records were unavailable at the consultation site; for others, consultation was impossible due to access restrictions to the building premises because of the COVID-19 pandemic (Figure 1).

### Data source

First, information on hospitalizations and deaths due to dengue in the municipality of Contagem was collected from the SUSFácil MG and Mortality Information System (*Sistema de Informação de Mortalidade* - SIM) databases. Next, a data extraction form was created to collect information from the municipality's death investigation records and from the medical records of hospitalizations of patients with dengue in Contagem who were admitted to the HMC (the only hospital in the city that provides care to patients through the SUS) and for whom death did not occur. The Contagem SMS is conducting a survey of all patients who died of dengue in the municipality. This is done by collecting data from medical records, interviews with family members, and data from medical records on care provided in primary healthcare units and emergency departments.

### Variables analyzed

A logistic regression model (univariate and multivariable) was used to determine the factors associated with death from dengue among Contagem residents, whose response variable was death from dengue (outcome). The independent (explanatory) variables analyzed were demographic variables (sex and age), the onset of symptoms, the start of treatment, hospitalization, case development, comorbidities (hypertension, asthma, chronic kidney disease, alcohol use, diabetes, smoking, heart disease, and other diseases), laboratory test results, treatment received during hospitalization, length of stay, time of onset of symptoms, and medical care/hospitalization.

### Statistical analysis

First, a preliminary analysis of the relationship between the independent variables and the response variable (cure x death from dengue) was performed using contingency tables. Only variables that had at least 80% of the total number of observations (i.e., at least 60 observations) were selected for analysis. Univariate logistic regressions were performed with explanatory and response variables to test for possible associations. Variables with a p < of 0.10 in the univariate analysis were selected to compose the initial (full) multivariable logistic model. Variables that did not meet the selection criteria (p < 0.10) but were considered important variables in the scientific literature related to dengue deaths were also evaluated. Variables with more than two categories were converted to dummy variables. Collinearity between variables was tested, and the variables that best explained dengue deaths were included in the analysis. OR was used as a measure of association. The "Backward Method" was used starting from the full model, and the variables that did not have a significance level of p < 0.05 in relation to the other variables that were adjusted were discarded sequentially. Variables with a statistical significance level of p < 0.05 and a significant OR according to a 95%CI remained in the final multivariable logistic model. The likelihood ratio test was used^13^. The performance of the model was evaluated by the area under the ROC curve. For a better interpretation of the continuous variables that remained in the final model, it was decided to calculate the OR by multiplying the regression coefficient (β) sometimes by five units of the continuous variable, sometimes by ten units, and then exponentiated according to the formulas "e^β x 5^" e "e^β x 10^", respectively^13^. Data analysis was performed using the statistical packages STATA version 15.0 (Stata Corp, College Station, Texas, United States) and Microsoft Excel 2013 (Washington, United States).

### Ethical aspects

This study was approved by the Ethics Committee of the *Universidade Federal de Minas Gerais*, with a certificate of presentation for ethical assessment corresponding to number 08527418.2.0000.5149. The project was also approved by the SMS of Contagem regarding access to medical records of dengue patients without a name or individual address.

## RESULTS

Age, myalgia, hemorrhage, headache, fatigue, abdominal pain, and dehydration had a significant p-value in univariate logistic regression analysis, demonstrating that they were potential factors associated with death from dengue. Regarding pre-existing diseases and alarm signs, diseases of the endocrine system, cardiovascular system, abdominal pain, and the presence of one or more alarm signs were considered potential risk factors for dengue cases in the evolution to death (Tables 1 and 2).

**TABLE 1:** Comparison of demographic characteristics, symptoms, and clinical variables in relation to dengue (cure x death), in 2016, in residents of the municipality of Contagem, State of Minas Gerais, Brazil.

	Dengue						
Variable	Cure n = 56		Death n =19		OR	95%CI	p
	n	%	n	%			
Age (years) (n =75)							
Median (IQR)	9 (6-12.5)		54 (35-77)		1.07	1.04-1.10	0.001*
Gender (n = 75)							
Males	29	51.8	5	26.3	3.00	0.95-9.47	0.060
Females	27	48.2	14	73.7			
Length of stay (n = 75)							
Median (IQR)	3(2-3)		3 (1-6)		1.13	0.96-1.33	0,134
Fever (n = 75)							
No	5	8.9	2	10.5	0.83	0.14-4.69	0.836
Yes	51	91.1	17	89.5			
Myalgia (n = 73)							
No	30	53.6	1	5.9	18.46	2.28-148.87	0.006*
Yes	26	46.4	16	94.1			
Exanthema (n = 64)							
No	23	41.1	4	50.0	0.69	0.15-3.07	0.634
Yes	33	58.9	4	50.0			
Plateletopenia (n = 73)							
No	6	10.9	5	27.8	0.31	0.08-1.20	0.093
Yes	49	89.1	13	72.2			
Hemoconcentration (n = 70)							
No	21	38.2	8	53.3	0.54	0.17-1.70	0.295
Yes	34	61.8	7	46.7			
Bleeds (n = 72)							
No	33	59.0	4	25.0	4.30	1.23-15.03	0.022*
Yes	23	41.0	12	75.0			
Vomiting (n = 70)							
No	30	53.6	6	42.9	1.53	0.47-5.01	0.475
Yes	26	46.4	8	57.1			
Headache (n = 71)							
No	36	65.5	5	31.3	4.16	1.26-13.76	0.019*
Yes	19	34.5	11	68.7			
Retroorbital pain (n = 67)							
No	45	81.8	7	58.3	3.21	0.84-12.23	0.087
Yes	10	18.2	5	41.7			
Prostration (n = 71)							
No	36	64.3	1	6.7	25.20	3.08-206.01	0.003*
Yes	20	35.7	14	93.3			
Abdominal pain (n = 65)							
No	44	78.6	3	33.3	7.33	1.59-33.72	0.010*
Yes	12	21.4	6	66.7			
Dehydration (n = 60)							
No	48	85.7	1	25.0	17.99	1.65-195.21	0.017*
Yes	8	14.3	3	75.0			

**n:** number of individuals. **OR:** Odds Ratio. **95%CI:** 95% confidence interval. I**QR:** interquartile range. *Significant p values.

**TABLE 2:** Comparison of pre-existing diseases and alarm signs in relation to dengue (cure x death) in 2016 in residents of the municipality of Contagem, State of Minas Gerais, Brazil.

	Dengue						
Variables	Cure n = 56		Death n =19		OR	95%CI	p
	n	%	n	%			
Pre-existing diseases							
Respiratory system (n = 75)							
No	52	92.9	18	94.7	0.72	0.07-6.89	0.777
Yes	4	7.1	1	5.3			
Endocrine system (n = 75)							
No	55	98.2	14	73.7	19.6	2.12-181.90	0.009*
Yes	1	1.8	5	26.3			
Cardiovascular System (n = 71)							
No	49	92.5	7	38.9	19.25	4.78-77.41	0.001*
Yes	4	7.5	11	61.1			
Obesity (n = 75)							
No	53	94.6	18	94.7	0.98	0.09-10.04	0.987
Yes	3	5.4	1	5.3			
**Signals and alarm**							
Alarm signals (n = 75)							
No	35	62.5	1	5.3	30.0	3.72-241.35	0.001*
Yes	21	37.5	18	94.7			
Abdominal pain (n = 75)							
No	53	94.6	14	76.7	6.30	1.34-29.66	0.020*
Yes	3	5.4	5	26.3			
Plateletopenia (n = 75)							
No	52	92.9	17	89.5	1.52	0.25-9.10	0.641
Yes	4	7.1	2	10.5			
Hepatomegaly (n = 75)							
No	54	96.4	19	100.0	1.00	---	---
Yes	2	3.6	0	0.0			
Hematocrit (n = 70)							
No	21	38.2	8	53.3	0.54	0.17-1.70	0.295
Yes	34	61.8	7	46.7			
Bleeds (n = 75)							
No	52	92.9	16	82.2	2.43	0.49-12.05	0.275
Yes	4	7.1	3	15.8			
Hypotension (n = 75)							
No	54	96.4	18	94.7	1.50	0.12-17.53	0.747
Yes	2	3.6	1	5.3			
Multiple alarm signals (n =75)							
No	50	89.3	12	63.2	4.86	1.37-17.12	0.014*
Yes	6	10.7	7	36.8			

**n:** number of individuals. **OR:** Odds Ratio. **95%CI:** 95% confidence interval. *Significant p values.

The variables associated with death from dengue in the final multivariable model (p < 0.05 and 95%CI) were age (OR = 1.07; 95%CI 1.03-1.11) and presence of bleeding (OR = 8.55; 95%CI 1.21-59.92). Each additional year of the patient's age increased the likelihood of that person dying from dengue. Stratified by 5 and 10 years, it can be concluded that with each fifth year of age, the probability increases 1.44-fold (OR = 1.44; 95%CI 1.21-1.71), and with each 10^th^ year of age, this probability increases to 2.07-fold (OR = 2.07; 95%CI 1.46-2.93). Patients with bleeding were 8.5 times more likely to die from dengue than patients who did not have bleeding (Table 3). The area under the ROC curve from the posttest adequacy assessment of the multivariable logistic regression model was 0.9353.

**TABLE 3:** Final multivariable model of factors associated with death from dengue in 2016, in residents of the municipality of Contagem, State of Minas Gerais, Brazil.

Variables	Regression Coefficient (β)	OR	p
	(95CI%)	(95%CI)	
Age	0.07 (0.04 - 0.10)	1.07 (1.03-1.11)	0.001
Bleeds	2.14 (0.19 - 4.09)	8.55 (1.21-59.92)	0.031

95%CI: 95% confidence interval. Log likelihood = -20.422686 / number of observations = 72/Pseudo R2 = 0.4645.

## DISCUSSION

In the municipality of Contagem, Minas Gerais, the southeast region of Brazil, there was a higher number of dengue deaths in 2016 than in previous years. The present case-control study on the factors associated with the occurrence of deaths is the first study known to analyze the prognostic factors associated with dengue-related deaths in this municipality. Age and hemorrhage were associated with deaths from the disease, highlighting the importance of adequate patient care to ensure access to appropriate diagnosis and clinical management by qualified health professionals, especially when affected individuals present with these characteristics.

The country's sharp increase in dengue fever over the years has been associated with the introduction and/or spread of one or more serotypes of the virus, in addition to the growing proportion of patients affected by the severe form of the disease. Between 2000 and 2015, the number of deaths caused by an arbovirus in Brazil increased by 639.0%, and the number of disability-adjusted life years (DALYs) lost increased by 266.1%^14^. These findings confirm that the severity of dengue epidemics in Brazil is increasing, as suggested by the reported increase in hospitalization rates^15^. In 2016, 609 dengue deaths were registered in the country, representing 6.8% of severe cases and alarm signs due to the disease, with the most severe forms of dengue occurring in the southeastern region^16^.

In Contagem, the number of deaths from dengue fever has caused concern among health authorities. In addition, studies have shown that the risk of severe is more significant when there is secondary infection with the dengue virus^17^ ^,^ ^18^. Due to the lack of information about secondary infections in the city, it was not possible to verify this condition in the patients evaluated in this study. However, it is known that different viral serotypes circulate in the municipality of Belo Horizonte, the capital of the state of Minas Gerais, a neighboring city of Contagem, suggesting that this dissemination also spreads to the municipality studied, increasing the likelihood of severe cases of the disease^18^.

In the present study, 25.4% of patients died due to dengue fever. Despite the variables of myalgia, hemorrhage, headache, prostration, abdominal pain, dehydration, diseases of the endocrine system, diseases of the cardiovascular system, and presence of one or more alarm signs, which were considered potential factors associated with death from the disease, only age and hemorrhage were significant. In this study, it was found that as age increased, the risk of death also increased. When the age group was stratified from five to five or from 10 to 10, the OR increased, indicating that the older the person, the greater the likelihood of dying from dengue. These findings corroborate those of other studies that have shown that age > 65 years and plasma extravasation were factors associated with death from dengue^19^ or even advanced age, hypertension, chronic kidney disease, and history of smoking, among other conditions^4^.

Advanced age was identified as a risk factor for severe forms of arbovirus. In 2015, the highest estimates of mortality rates in Brazil were recorded in children under one year of age and in those over 65 years of age^14^. In Minas Gerais, a study found that after 2011, the year DENV-4 was introduced, the highest mortality rates occurred in patients over 50 years of age due to the difficulty of treating the disease in a population with an increased number of comorbidities^20^.

The susceptibility of children under 15 years of age, particularly those under one year of age, has been associated with the severe form of the disease^4^. Several authors have reported this predisposition since the 1960s^4^. In the late 1980s, this susceptibility was explained by the progressive decline of maternal antibodies in children under one year of age; instead of protection, these antibodies would facilitate infection by DENV^21^. Additionally, in Brazil, it was found that the highest DALY rates per 100,000 population for dengue were in children under one year of age in 2000 and 2015, with a 358.2% increase between years^14^. The same study found a decrease in DALY rates in the one-to-four-year age group, an increase in the five-to-nine-year age group, and stabilization of the indicator in the later age groups^14^.

A historical cohort study conducted in the state of Minas Gerais in southeastern Brazil indicated that factors associated with death from dengue fever included age greater than 65 years, blood plasma leakage, and residence in a municipality with a population of less than 100,000^12^. Recent findings have shown that dengue mortality in São Paulo increases with age, peaking at the age of 70 years or older (1.41 deaths per 100,000 population-years)^22^. In addition, the distribution of deaths was heterogeneous in space and time^22^.

People with hemorrhagic manifestations were 8.5 times more likely to die from dengue than patients who did not have bleeding in Contagem. In Brazil, a study was conducted in 2007 in the city of Rio de Janeiro that investigated deaths from dengue and found that 69.3% of patients had hemorrhagic manifestations (gastrointestinal bleeding, 42.3%; petechiae, 26.1%; epistaxis, 19.2%; gum bleeding, 11.5%)^23^.

There is a great deal of discussion about the reasons for the development of bleeding in dengue patients, such as vascular factors, imbalances in coagulation and fibrinolysis (the process by which a fibrin clot, a blood coagulation product, is destroyed), and thrombocytopenia (characterized by a low platelet count)^24^ ^,^ ^25^. It has also been argued that severe dengue is related to plasma leakage leading to shock^4^. Prolonged hypoperfusion has also been cited as a factor in bleeding in patients with severe dengue fever because it leads to metabolic acidosis, multiorgan failure, and disseminated intravascular coagulation^4^.

This study provides important information about the factors associated with dengue deaths in the community of Contagem. However, this method has some limitations. It is noteworthy that the records analyzed lacked information on clinical evolution and laboratory test results.

The lack of information on laboratory tests is because patients treated in the emergency room remain there until they are admitted to the Municipal Hospital of Contagem. During the dengue epidemic, the hospitals had considerable capacity. Thus, the waiting time for patients to be admitted to a hospital varies from two to four days. When they are admitted to the hospital, they stay only a short time because the severity phase of the disease has ended. Because of the short length of stay in the hospital, patients were discharged before the test results were available, and consequently, they were not included in the medical record. Therefore, no information on the test results was found in the medical records.

In addition, access to the medical records was interrupted because of the COVID-19 pandemic outbreak. For this reason, estimates were less accurate and confidence intervals wider because of the small number of cases.

Another point worth highlighting is the heterogeneity of the sample in the present study with respect to the age group of the study participants. It should be considered that the municipality of Contagem has a unique municipal hospital, and the data presented in this study refer to an epidemic period. In addition, due to the lack of data, it was not possible to demonstrate the association between deaths and the predominant circulating dengue serotype in the present study. Secondary infections^26^ or even those caused by DENV-1 and DENV-2^27^ can lead to death regardless of the age of the affected person. More detailed studies in the municipality of Minas Gerais and a larger number of observations would be needed to deepen these explanations.

Early detection and investigation of dengue-related deaths can be an indicator for health managers in organizing health services and training professionals who directly care for patients. New studies should be conducted that consider the stays of patients in emergency care units because it has been observed that most patients were hospitalized in these health facilities before admission.
